# Supplementary material for: Salivary Diagnosis of Dental Caries: A Systematic Review
Source: Curr Issues Mol Biol. 2024 May 2;46(5):4234–50. doi: 10.3390/cimb46050258 (PMC11120503; doi:10.3390/cimb46050258)
Supplement: Supplementary file 1 [file cimb-46-00258-s001.zip › cimb-2943736-supplementary.pdf]

Table S1 "JBJ Critical appraisal Checklist" for case control studies

Question	Explanation
1. Were the groups comparable other than the presence of disease in cases or the absence of disease in cases or the absence of disease in controls?	Comparability was assigned if cases and controls did not have systemic diseases, oral diseases excluding caries, did not smoke, were not alcohol and / or drug users
2. Were cases and controls matched appropriately?	Matching was assigned if data regarding age and sex of subjects were similar, as well as the sample size.
3. Were the same criteria used for identification of cases and controls?	Studies that did not use clinical and/or radiographic diagnostic methods to confirm the presence (cases) or absence (controls) of dental caries were considered to have an high risk of bias (e.g., a rapid clinical evaluation using an explorer performed by only one operator).
4. Was the exposure measured in a standard, valid and reliable way?	Not applicable
5. Was the exposure measured in the same way for cases and controls?	Not applicable
6. Were confounding factors identified?	Studies that did not mention to have investigate confounding factor or did not list these in the paper were considered to have an high risk of bias.
7. Were strategies to deal with confounding factors stated?	Studies that mentioned confounding factors and that use appropriated statistical analysis (for example a multivariate regression analysis) to normalizing these, were considered to have a low risk of bias.
8. Were outcomes assessed on a standard, valid and reliable way for cases and controls?	If the outcomes were assessed using a standard diagnostic criterion (e.g., DMTF index, ICDAS criteria), the study were considered to have a low risk of bias.
9. Was the exposure period of interest long enough to be meaningful?	Not applicable
10. Was appropriate statistical analysis used?	If the study described clearly the valid statistical analysis used, were considered to have a low risk of bias.

Table S2 "JBJ Critical appraisal Checklist" for cross sectional studies

Question	Explanation
1. Were the criteria for inclusion in the sample clearly defined?	Studies that did not specify the inclusion/exclusion criteria, and not mention any other information about the patients (e.g, presence of systemic diseases, risk factors) were considered to have an high risk of bias.
2. Were the study subjects and the settings described in detail?	Studies that did not provide a clear description of the population from which the study participants were selected or recruited (including demographics, location, and time period) were considered to have an high risk of bias.
3. Was the exposure measured in a valid and reliable way?	Not applicable
4. Were the objective, standard criteria used for measurement of the condition?	Studies that did not use clinical and/or radiographic diagnostic methods to confirm the presence (cases) or absence (controls) of dental caries were considered to have an high risk of bias (e.g., a rapid clinical evaluation using an explorer performed by only one operator).
5. Were confounding factors identified?	Studies that did not mention to have investigate confounding factor or did not list these in the paper were considered to have an high risk of bias.
6. Were strategies to deal with confounding factors stated?	Studies that mentioned confounding factors and that use appropriated statistical analysis (for example a multivariate regression analysis) to normalizing these, were considered to have a low risk of bias.
7. Were the outcomes measured in a valid and reliable way?	If the outcomes were measured using a standard diagnostic criterion (e.g., DMTF index, ICDAS criteria), the study were considered to have a low risk of bias.
8. Was appropriate statistical analysis used?	If the study described clearly the valid statistical analysis used, were considered to have a low risk of bias.

Table S3 Papers excluded and the reasons for exclusion

	References of the excluded paper	Reason for the exclusion after full text reading
**1.**	Ademe D, Admassu D, Balakrishnan S. Analysis of salivary level Lactobacillus spp. And associated factors as determinants of dental caries amongst primary school children in Harar town, eastern Ethiopia. BMC Pediatrics. 2020;20(1).	• Different aim<br>• Study on the evaluation of deciduous teeth
**2.**	AlMaummar M, AlThabit HO, Pani S. The impact of dental treatment and age on salivary cortisol and alpha-amylase levels of patients with varying degrees of dental anxiety. BMC Oral Health. 2019 Sep 6;19(1):211. doi: 10.1186/s12903-019-0901-7. PMID: 31492133; PMCID: PMC6731601.	• Different aim
**3.**	Anjomshoaa I, Briseño-Ruiz J, Deeley K, Poletta FA, Mereb JC, Leite AL, Barreta PA, Silva TL, Dizak P, Ruff T, Patir A, Koruyucu M, Abbasoğlu Z, Casado PL, Brown A, Zaky SH, Bayram M, Küchler EC, Cooper ME, Liu K, Marazita ML, Tanboğa İ, Granjeiro JM, Seymen F, Castilla EE, Orioli IM, Sfeir C, Owyang H, Buzalaf MA, Vieira AR. Aquaporin 5 Interacts with Fluoride and Possibly Protects against Caries. PLoS One. 2015 Dec 2;10(12):e0143068. doi: 10.1371/journal.pone.0143068. PMID: 26630491; PMCID: PMC4668048.	• Different aim
**4.**	Belstrøm D, Fiehn NE, Nielsen CH, Holmstrup P, Kirkby N, Klepac-Ceraj V, et al. Altered bacterial profiles in saliva from adults with caries lesions: a case-cohort study. Caries Res. 2014;48(5):368-75. PubMed PMID: 24643218. Epub 2014/03/20. eng.	• Different aim
**5.**	Belstrøm D, Holmstrup P, Bardow A, Kokaras A, Fiehn NE, Paster BJ. Comparative analysis of bacterial profiles in unstimulated and stimulated saliva samples. Journal of Oral Microbiology. 2016;8(1).	• Different aim
**6.**	Belstrom D, Holmstrup P, Fiehn NE, Kirkby N, Kokaras A, Paster BJ, et al. Salivary microbiota in individuals with different levels of caries experience. Journal of Oral Microbiology. 2017;9. PubMed PMID: WOS:000396759900001.	• Different aim
**7.**	Børsting T, Venkatraman V, Fagerhaug TN, Skeie MS, Stafne SN, Feuerherm AJ, Sen A. Systematic assessment of salivary inflammatory markers and dental caries in children: an exploratory study. Acta Odontol Scand. 2022 Jul;80(5):338-345. doi: 10.1080/00016357.2021.2011400. Epub 2021 Dec 7. PMID: 34875210.	• Study on the evaluation of deciduous teeth
**8.**	Bruvo M, Moe D, Kirkeby S, Vorum H, Bardow A. Individual variations in protective effects of experimentally formed salivary pellicles. Caries Res. 2009;43(3):163-70. PubMed PMID: 19390190. Epub 2009/04/25. eng.	• Different aim
**9.**	Campus G, Lumbau A, Bachisio SL. Caries experience and streptococci and lactobacilli salivary levels in 6-8-year-old Sardinians. Int J Paediatr Dent. 2000 Dec;10(4):306-12. PubMed PMID: 11310244. Epub 2001/04/20. eng.	• Different aim<br>• Study on the evaluation of deciduous teeth
**10.**	Castagnola M, Picciotti PM, Messana I, Fanali C, Fiorita A, Cabras T, et al. Potential applica- tions of human saliva as diagnostic fluid. Acta Otorhinolaryngol Ital. 2011;31(6):347–57.	• Review

**11.**	Chen W, Jiang Q, Yan GW, Yang DQ. The oral microbiome and salivary proteins influence caries in children aged 6 to 8 years. Bmc Oral Health. 2020 Oct;20(1). PubMed PMID: WOS:000590554800001.	• Study on the evaluation of deciduous teeth
**12.**	Colombo NH, Pereira JA, Da Silva MER, Ribas LFF, Parisotto TM, Mattos-Graner RDO, et al. Relationship between the IgA antibody response against Streptococcus mutans GbpB and severity of dental caries in childhood. Archives of Oral Biology. 2016;67:22-7.	• Study on the evaluation of deciduous teeth
**13.**	Dashper SG, Mitchell HL, Cao KAL, Carpenter L, Gussy MG, Calache H, et al. Temporal development of the oral microbiome and prediction of early childhood caries. Scientific Reports. 2019 Dec;9. PubMed PMID: WOS:000508915300003.	• Different aim<br>• Study on the evaluation of deciduous teeth
**14.**	Davis E, Bakulski KM, Goodrich JM, Peterson KE, Marazita ML, Foxman B. Low levels of salivary metals, oral microbiome composition and dental decay. Sci Rep. 2020 Sep 4;10(1):14640. PubMed PMID: 32887894. PMCID: PMC7474081. Epub 2020/09/06. eng.	• Different aim
**15.**	Dawes C. The effects of exercise on protein and electrolyte secretion in parotid saliva. J Physi- ol. 1981;320(1):139–48.	• Different aim
**16.**	de Andrade CM, Galvão-Moreira LV, de Oliveira JFF, Bomfim MRQ, Monteiro SG, Figueiredo PMS, Branco-de-Almeida LS. Salivary biomarkers for caries susceptibility and mental stress in individuals with facial pain. Cranio. 2021 May;39(3):231-237. doi: 10.1080/08869634.2019.1607445. Epub 2019 May 1. PMID: 31043147.	• Different aim
**17.**	de Sousa Né YG, Lima WF, Mendes PFS, Baia-da-Silva DC, Bittencourt LO, Nascimento PC, de Souza-Rodrigues RD, Paranhos LR, Martins-Júnior PA, Lima RR. Dental Caries and Salivary Oxidative Stress: Global Scientific Research Landscape. Antioxidants (Basel). 2023 Jan 31;12(2):330. doi: 10.3390/antiox12020330. PMID: 36829890; PMCID: PMC9952432.	• Review
**18.**	Dina G. Moussa, Paras Ahmad, Tamer A. Mansour, Walter L. Siqueira, Current State and Challenges of the Global Outcomes of Dental Caries Research in the Meta-Omics Era, Frontiers in Cellular and Infection Microbiology, 10.3389/fcimb.2022.887907, **12**, (2022).	• Review
**19.**	Fernando S, Tadakamadla S, Kroon J, Lalloo R, Johnson NW. Predicting dental caries increment using salivary biomarkers in a remote Indigenous Australian child population. BMC Oral Health. 2021 Jul 23;21(1):372. doi: 10.1186/s12903-021-01702-0. PMID: 34301228; PMCID: PMC8305904.	• Study on the evaluation of deciduous teeth
**20.**	Gao X, Jiang S, Koh D, Hsu CY. Salivary biomarkers for dental caries. Periodontol 2000. 2016 Feb;70(1):128-41. doi: 10.1111/prd.12100. PMID: 26662487.	• Review
**21.**	Golpasand Hagh L, Zakavi F, Ansarifar S, Ghase- mzadeh O, Solgi G. Association of dental car- ies and salivary sIgA with tobacco smoking.	• Different aim
**22.**	Grootveld M, Page G, Bhogadia M, Hunwin K, Edgar M. Updates and Original Case Studies Focused on the NMR-Linked Metabolomics Analysis of Human Oral Fluids Part III: Implementations for the Diagnosis of Non-Cancerous Disorders, Both Oral and Systemic. Metabolites. 2023 Jan 1;13(1):66. doi: 10.3390/metabo13010066. PMID: 36676991; PMCID: PMC9864626.	• Different aim
**23.**	Hertel S, Hannig C, Sterzenbach T. The abundance of lysozyme, lactoferrin and cystatin S in the enamel pellicle of children - Potential	• Different aim

	biomarkers for caries? Arch Oral Biol. 2023 Feb;146:105598. doi: 10.1016/j.archoralbio.2022.105598. Epub 2022 Dec 6. PMID: 36525870.	• Study on the evaluation of deciduous teeth
**24.**	Hong SW, Seo DG, Baik JE, Cho K, Yun CH, Han SH. Differential profiles of salivary proteins with affinity to Streptococcus mutans lipoteichoic acid in caries-free and caries-positive human subjects. Mol Oral Microbiol. 2014 Oct;29(5):208-18. doi: 10.1111/omi.12057. Epub 2014 Jul 28. PMID: 24848678.	• Different aim
**25.**	Huang B, Cvitkovitch DG, Santerre JP, Finer Y. Biodegradation of resin-dentin interfaces is dependent on the restorative material, mode of adhesion, esterase or MMP inhibition. Dent Mater. 2018 Sep;34(9):1253-1262. doi: 10.1016/j.dental.2018.05.008. Epub 2018 May 19. PMID: 29789163.	• Different aim
**26.**	Jagadesh Babu B, Venugopal Reddy N, Thimma Reddy B, Daneswari V, Puppala N. Compari- tive evaluation of salivary IgA levels and den- tal caries in obese and non-obese children. Int J Adv Res. 2017;5(1):766–72.	• Different aim<br>• Study on the evaluation of deciduous teeth
**27.**	Jonasson A, Eriksson C, Jenkinson HF, Källestål C, Johansson I, Strömberg N. Innate immunity glycoprotein gp-340 variants may modulate human susceptibility to dental caries. BMC Infect Dis. 2007 Jun 11;7:57. PubMed PMID: 17562017. PMCID: PMC1894970. Epub 2007/06/15. eng.	• Study on the evaluation of deciduous teeth
**28.**	Kho HS. Oral epithelial MUC1 and oral health. Oral Dis. 2018 Mar;24(1-2):19-21. doi: 10.1111/odi.12713. PMID: 29480594.	• Different aim
**29.**	Kimoto M, Iwai S, Maeda T, Yura Y, Fernley RT, Ogawa Y. Carbonic anhydrase VI in the mouse nasal gland. J Histochem Cytochem. 2004;52(8):1057–62.	• No human study population
**30.**	Lalloo R, Tadakamadla SK, Kroon J, Tut O, Kularatna S, Boase R, Kapellas K, Gilchrist D, Cobbledick E, Rogers J, Johnson NW. Salivary characteristics and dental caries experience in remote Indigenous children in Australia: a cross-sectional study. BMC Oral Health. 2019 Jan 17;19(1):21. doi: 10.1186/s12903-018-0692-2. PMID: 30654791; PMCID: PMC6337781.	• Study on the evaluation of deciduous teeth
**31.**	Leinonen J, Kivelä J, Parkkila S, Parkkila AK, Ra- janiemi H. Salivary carbonic anhydrase isoen- zyme VI is located in the human enamel pellicle. Caries Res. 1999;33(3):185–90.	• Different aim
**32.**	Li S, Huang S, Guo Y, Zhang Y, Zhang L, Li F, Tan K, Lu J, Chen Z, Guo Q, Tang Y, Teng F, Yang F. Geographic Variation Did Not Affect the Predictive Power of Salivary Microbiota for Caries in Children With Mixed Dentition. Front Cell Infect Microbiol. 2021 Jun 18;11:680288. doi: 10.3389/fcimb.2021.680288. PMID: 34222048; PMCID: PMC8250437.	• Study on the evaluation of deciduous teeth
**33.**	Li X, Wang L, Nunes DP, Troxler RF, Offner GD. Pro-inflammatory cytokines up-regulate MUC1 gene expression in oral epithelial cells.	• Different aim
**34.**	Lira-Junior R, Åkerman S, Gustafsson A, Klinge B, Boström EA. Colony stimulating factor-1 in saliva in relation to age, smoking, and oral and systemic diseases. Sci Rep. 2017 Aug 4;7(1):7280. doi: 10.1038/s41598-017-07698-4. PMID: 28779164; PMCID: PMC5544729.	• Different aim
**35.**	Lorenzo-Pouso AI, Pérez-Sayáns M, Bravo SB, López-Jornet P, García-Vence M, Alonso-Sampedro M, Carballo J, García-García A. Protein-Based Salivary Profiles as Novel Biomarkers for Oral Diseases. Dis Markers. 2018 Nov 7;2018:6141845. doi: 10.1155/2018/6141845. PMID: 30524521; PMCID: PMC6247606.	• Review

**36.**	Luo JY, Wang YF, Wang K, Jiang WT, Li XW, Zhang LL. Comparative proteomic analysis on acquired enamel pellicle at two time points in caries-susceptible and caries-free subjects. Journal of Dentistry. 2020 Mar;94. PubMed PMID: WOS:000518851100002.	• Different aim
**37.**	Marquezin MCS, Scudine KGO, Lamy E, Finassi CM, Carreira L, Segura WD, Rasera I Jr, Pessotti ER, Castelo PM. Impact of gastroplasty on salivary characteristics, dental health status and oral sensory aspects: A controlled clinical study. J Oral Rehabil. 2022 Oct;49(10):1002-1011. doi: 10.1111/joor.13353. Epub 2022 Jul 10. PMID: 35751503.	• Different aim
**38.**	McAuley JL, Linden SK, Png CW, King RM, Pen- nington HL, Gendler SJ, et al. MUC1 cell sur- face mucin is a critical element of the mucosal barrier to infection. J Clin Invest. 2007;117(8)	• Different aim
**39.**	Morou-Bermudez E, Loza-Herrero MA, Garcia-Rivas V, Suarez-Perez E, Billings RJ. Oral bacterial acid–base metabolism in caries screening: A proof-of-concept study. JDR Clinical and Translational Research. 2017;2(2):132-41.	• Different aim
**40.**	Moussa DG, Ahmad P, Mansour TA, Siqueira WL. Current state and challenges of the glob- al outcomes of dental caries research in the meta-omics era. Front Cell Infect Microbiol. 2022;12:887907.	• Review
**41.**	Nakas E, Zukanovic A. The prevalence of cariogenic salivary micro-organisms in children of various ages. Bosnian Journal of Basic Medical Sciences. 2007 May;7(2):166-70. PubMed PMID: WOS:000258296300015.	• Study on the evaluation of deciduous teeth
**42.**	Neves AB, Lobo LA, Pinto KC, Pires ES, Requejo MEP, Maia LC, et al. Comparison between clinical aspects and salivary microbial profile of children with and without early childhood caries: A preliminary study. Journal of Clinical Pediatric Dentistry. 2015;39(3):209-14.	• Study on the evaluation of deciduous teeth
**43.**	Nishikawara F, Katsumura S, Ando A, Tamaki Y, Nakamura Y, Sato K, et al. Correlation of cariogenic bacteria and dental caries in adults. J Oral Sci. 2006 Dec;48(4):245-51. PubMed PMID: 17220624. Epub 2007/01/16. eng.	• Different aim
**44.**	Nomura R, Nakano K, Taniguchi N, Lapirattanakul J, Nemoto H, Gronroos L, et al. Molecular and clinical analyses of the gene encoding the collagen-binding adhesin of Streptococcus mutans. Journal of Medical Microbiology. 2009 Apr;58(4):469-75. PubMed PMID: WOS:000264908600013.	• Different aim
**45.**	Offner GD, Troxler RF. Heterogeneity of high- molecular-weight human salivary mucins. Adv Dent Res. 2000;14(1):69–75.	• Review
**46.**	Oktysyuk Y, Rozhko MM, Bazalytska O. Correction of mineral methabolism of saliva in children affected by dental caries. Pharmacia. 2017;64(3):40-5.	• Study on the evaluation of deciduous teeth
**47.**	Ollila PS, Larmas MA. Long-term predictive value of salivary microbial diagnostic tests in children. Eur Arch Paediatr Dent. 2008 Mar;9(1):25-30. PubMed PMID: 18328235. Epub 2008/03/11. eng.	• Different aim<br>• Study on the evaluation of deciduous teeth
**48.**	Pal S, Mitra M, Mishra J, Saha S, Bhattacharya B. Correlation of total salivary secretory immu- noglobulin A (SIgA) and mutans specific SIgA in children having different caries status. Indian Soc Pedod Prev Dent. 2013;31(4):270– 4.	• Study on the evaluation of deciduous teeth
**49.**	Panagiotou E, Agouropoulos A, Vadiakas G, Pervanidou P, Chouliaras G, Kanaka-Gantenbein C. Oral health of overweight and obese children and adolescents: a comparative study with a	• Different aim

	multivariate analysis of risk indicators. Eur Arch Paediatr Dent. 2021 Oct;22(5):861-868. doi: 10.1007/s40368-021-00643-0. Epub 2021 Jun 11. PMID: 34117610.	
**50.**	Pang L, Wang K, Tao Y, Zhi Q, Zhang J, Lin H. A New Model for Caries Risk Prediction in Teenagers Using a Machine Learning Algorithm Based on Environmental and Genetic Factors. Front Genet. 2021 Mar 11;12:636867. doi: 10.3389/fgene.2021.636867. PMID: 33777105; PMCID: PMC7990890.	• Different aim
**51.**	Pannu P, Gambhir R, Sujlana A. Correlation between the salivary Streptococcus mutans levels and dental caries experience in adult population of Chandigarh, India. European Journal of Dentistry. 2013;7(2):191-5.	• Different aim
**52.**	Parisotto TM, King WF, Duque C, Mattos-Graner RO, Steiner-Oliveira C, Nobre-Dos-Santos M, et al. Immunological and microbiologic changes during caries development in young children. Caries Res. 2011;45(4):377-85. PubMed PMID: 21822016. Epub 2011/08/09. eng.	• Study on the evaluation of deciduous teeth
**53.**	Patidar D, Sogi S, Singh V, Shinu P, Loomba A, Patidar DC. Salivary levels of Streptococcus mutans and Streptococcus sanguinis in early childhood caries: An in vivo study. J Indian Soc Pedod Prev Dent. 2018 Oct-Dec;36(4):386-90. PubMed PMID: 30324930. Epub 2018/10/17. eng.	• Different aim<br>• Study on the evaluation of deciduous teeth
**54.**	Pellegrini G, Maddalone M, Malvezzi M, Toma M, Del Fabbro M, Canciani E, Dellavia C. sCD14 Level in Saliva of Children and Adolescents with and without Dental Caries, a Hurdle Model. Children (Basel). 2021 Aug 4;8(8):679. doi: 10.3390/children8080679. PMID: 34438570; PMCID: PMC8394623.	• Study on the evaluation of deciduous teeth
**55.**	Perez VA, Mangum JE, Hubbard MJ. Pathogenesis of Molar Hypomineralisation: Aged Albumin Demarcates Chalky Regions of Hypomineralised Enamel. Front Physiol. 2020 Sep 30;11:579015. doi: 10.3389/fphys.2020.579015. PMID: 33101060; PMCID: PMC7556231.	• Different aim
**56.**	Petersen PE, Bourgeois D, Ogawa H, Estupinan- Day S, Ndiaye C. The global burden of oral diseases and risks to oral health. Bull World Health Organ. 2005;83:661–9.	• Different aim
**57.**	Phattarataratip E, Olson B, Broffitt B, Qian F, Brogden KA, Drake DR, et al. Streptococcus mutans strains recovered from caries-active or caries-free individuals differ in sensitivity to host antimicrobial peptides. Mol Oral Microbiol. 2011 Jun;26(3):187-99. PubMed PMID: 21545696. PMCID: PMC3092152. Epub 2011/05/07. eng.	• Study on the evaluation of deciduous teeth
**58.**	Pineda AG-A, Pérez AG, García-Godoy F. Salivary parameters and oral health status amongst adolescents in Mexico. *BMC Oral Health*. 2020;**20**(1):1–7. doi: 10.1186/s12903-019-0991-2.	• Different aim
**59.**	Piwat S, Teanpaisan R, Thitasomakul S, Thearmontree A, Dahlén G. Lactobacillus species and genotypes associated with dental caries in Thai preschool children. Mol Oral Microbiol. 2010 Apr;25(2):157-64. PubMed PMID: 20331803. Epub 2010/03/25. eng.	• Different aim:<br>• Study on the evaluation of deciduous teeth
**60.**	Pramanik R, Osailan SM, Challacombe SJ, Urqu- hart D, Proctor GB. Protein and mucin reten- tion on oral mucosal surfaces in dry mouth patients. Eur J Oral Sci. 2010;118(3):245–53.	• Different aim
**61.**	Preethi BP, Reshma D, Anand P. Evaluation of flow rate, pH, buffering capacity, calcium, to- tal proteins and total antioxidant capacity lev- els of saliva in caries free and caries active chil- dren: an in vivo study. Indian J Clin Biochem. 2010;25(4):425–8.	• Different aim<br>• Study on the evaluation of deciduous teeth

**62.**	Preethi BP, Reshma D, Anand P. Evaluation of flow rate, pH, buffering capacity, calcium, to- tal proteins and total antioxidant capacity lev- els of saliva in caries free and caries active children: an in vivo study. Indian J Clin Biochem. 2010;25(4):425–8	• Different aim<br>• Study on the evaluation of deciduous teeth
**63.**	Priya PR, Asokan S, Karthick K, Reddy NV, Rao VA. Effect of dental treatments on salivary immunoglobulin A of children with and with- out dental caries: a comparative study. Indian J Dent Res. 2013;24(3):394.	• Study on the evaluation of deciduous teeth
**64.**	Pyati SA, Naveen Kumar R, Kumar V, Praveen Kumar NH, Parveen Reddy KM. Salivary flow rate, pH, buffering capacity, total protein, ox- idative stress and antioxidant capacity in chil- dren with and without dental caries. J Clin Pediatr Dent. 2018;42(6):445–9.	• Different aim<br>• Study on the evaluation of deciduous teeth
**65.**	Raviraj GA, Bhat KG, Kugaji MS, Kumbar VM, Hooli A. Study of microbial diversity in saliva and plaque samples from caries-free and caries-affected children using denaturing gradient gel electrophoresis. J Indian Soc Pedod Prev Dent. 2018 Oct-Dec;36(4):396-401. PubMed PMID: 30324932. Epub 2018/10/17. eng.	• Different aim<br>• Study on the evaluation of deciduous teeth
**66.**	Razi MA, Qamar S, Singhal A, Mahajan A, Sid- diqui S, Mohina Minz RS. Role of natural sal- ivary defenses in the maintenance of healthy oral microbiota in children and adolescents. J Fam Med Prim Care. 2020;9(3):1603–7.	• Different aim<br>• Study on the evaluation of deciduous teeth
**67.**	Rodis OMM, Matsumura S, Kariya N, Okazaki Y, Ogata S, Reissmann DR. Culture-based PCR analysis of plaque samples of Japanese school children to assess the presence of six common cariogenic bacteria and its association with caries risk. Molecular and Cellular Probes. 2009 Dec;23(6):259-63. PubMed PMID: WOS:000272069100001.	• Different aim<br>• Study on the evaluation of deciduous teeth
**68.**	Rosa N, Campos B, Esteves AC, Duarte AS, Correia MJ, Silva RM, Barros M. Tracking the functional meaning of the human oral-microbiome protein-protein interactions. Adv Protein Chem Struct Biol. 2020;121:199-235. doi: 10.1016/bs.apcsb.2019.11.014. Epub 2020 Jan 16. PMID: 32312422.	• Different aim
**69.**	Rupf S, Laczny CC, Galata V, Backes C, Keller A, Umanskaya N, et al. Comparison of initial oral microbiomes of young adults with and without cavitated dentin caries lesions using an in situ biofilm model. Sci Rep. 2018 Sep 18;8(1):14010. PubMed PMID: 30228377. PMCID: PMC6143549. Epub 2018/09/20. eng.	• Different aim
**70.**	Sakeenabi B, Hiremath SS. Dental caries experience and salivary streptococcus mutans,lactobacilli scores, salivary flow rate and salivary buffering capacity among 6 year old Indian school children. Journal of Clinical and Experimental Dentistry. 2011;3(5):e412-e7.	• Different aim
**71.**	Sandhya, Gayathri R, Vishnupriya V. Resistance gene screening of pathogens from saliva sample of caries patients. International Journal of Pharmaceutical Sciences Review and Research. 2016;39(2):112-5.	• Different aim
**72.**	Sanz M, Beighton D, Curtis MA, Cury JA, Dige I, Dommisch H, et al. Role of microbial biofilms in the maintenance of oral health and in the development of dental caries and periodontal diseases. Consensus report of group 1 of the Joint EFP/ORCA workshop on the boundaries between caries and periodontal disease. Journal of Clinical Periodontology. 2017 Mar;44:S5-S11. PubMed PMID: WOS:000418237200001.	• Different aim
**73.**	Șaramet V, Meleșcanu-Imre M, Țâncu AMC, Albu CC, Ripszky-Totan A, Pantea M. Molecular Interactions between Saliva and Dental	• Different aim

	Composites Resins: A Way Forward. Materials (Basel). 2021 May 13;14(10):2537. doi: 10.3390/ma14102537. PMID: 34068320; PMCID: PMC8153278.	
**74.**	Sejdini M, Begzati A, Salihu S, Krasniqi S, Berisha N, Aliu N. The Role and Impact of Salivary Zn Levels on Dental Caries. International Journal of Dentistry. 2018;2018.	• Different aim
**75.**	Sejdini M, Berisha N, Citaku E. Determination and influence of saliva calcium and magnesium in children with different intensity of caries. Journal of International Dental and Medical Research. 2018;11(1):51-6.	• Different aim:
**76.**	Seki M, Karakama F, Terajima T, Ichikawa Y, Ozaki T, Yoshida S, et al. Evaluation of mutans streptococci in plaque and saliva: correlation with caries development in preschool children. J Dent. 2003 May;31(4):283-90. PubMed PMID: 12735923. Epub 2003/05/09. eng.	• Different aim<br>• Study on the evaluation of deciduous teeth
**77.**	Selwitz RH, Ismail AI, Pitts NB. Dental caries. Lancet. 2007;369(9555):51–9.	• Review
**78.**	Seredin P, Goloshchapov D, Ippolitov Y, Vongsvivut J. Comparative analysis of dentine and gingival fluid molecular composition and protein conformations during development of dentine caries: A pilot study. Vibrational Spectroscopy. 2020 May;108. PubMed PMID: WOS:000534552000004.	• Different aim
**79.**	Shahrabi M, Nikfarjam J, Alikhani A, Akhoundi N, Ashtiani M, Seraj B. A comparison of salivary calcium, phosphate, and alkaline phosphatase in children with severe, moderate caries, and caries free in Tehran's kindergartens. J Indian Soc Pedod Prev Dent. 2008 Jun;26(2):74-7. PubMed PMID: 18603733. Epub 2008/07/08. eng.	• Different aim
**80.**	Shi C, Cai L, Xun Z, Zheng S, Shao F, Wang B, et al. Metagenomic analysis of the salivary microbiota in patients with caries, periodontitis and comorbid diseases. Journal of Dental Sciences. 2020.	• Different aim
**81.**	Shimotoyodome A, Kobayashi H, Tokimitsu I, Hase T, Inoue T, Matsukubo T, et al. Saliva- promoted adhesion of *Streptococcus mutans* MT8148 associates with dental plaque and caries experience. Caries Res. 2007;41(3): 212–8.	• Different aim
**82.**	Shu M, Morou-Bermudez E, Suarez-Perez E, Rivera-Miranda C, Browngardt CM, Chen YYM, et al. The relationship between dental caries status and dental plaque urease activity. Oral Microbiology and Immunology. 2007 Feb;22(1):61-6. PubMed PMID: WOS:000243479100010.	• Different aim
**83.**	Silbereisen A, Lira-Junior R, Åkerman S, Klinge B, Boström EA, Bostanci N. Association of salivary TREM-1 and PGLYRP1 inflammatory markers with non-communicable diseases. J Clin Periodontol. 2023 Nov;50(11):1467-1475. doi: 10.1111/jcpe.13858. Epub 2023 Jul 31. PMID: 37524498.	• Different aim
**84.**	Siqueira WL, Custodio W, McDonald EE. New insights into the composition and functions of the acquired enamel pellicle. J Dent Res. 2012 Dec;91(12):1110-8. PubMed PMID: 23018818. Epub 2012/09/29. eng.	• Different aim
**85.**	Siqueira WL, Dawes C. The salivary proteome: challenges and perspectives. Proteom Clin Appl. 2011;5(11–12):575–9.	• Review
**86.**	Siqueira WL, Helmerhorst EJ, Zhang W, Salih E, Oppenheim FG. Acquired enamel pellicle and its potential role in oral diagnostics. Ann N Y Acad Sci. 2007 Mar;1098:504-9. PubMed PMID: 17435160. Epub 2007/04/17. eng.	• Different aim

**87.**	Smith SI, Aweh AJ, Coker AO, Savage KO, Abosede DA, Oyedeji KS. Lactobacilli in human dental caries and saliva. Microbios. 2001;105(411):77-85. PubMed PMID: 11393751. Epub 2001/06/08. eng.	• Different aim
**88.**	Soesilawati P, Notopuro H, Yuliati Y, Ariani MD, Alwino Bayu Firdauzy M. The role of salivary sIgA as protection for dental caries activity in Indonesian children. Clin Cosmet Investig Dent. 2019;11:291–5.	• Study on the evaluation of deciduous teeth
**89.**	Sounah SA, Madfa AA. Correlation between dental caries experience and the level of Streptococcus mutans and lactobacilli in saliva and carious teeth in a Yemeni adult population. BMC Res Notes. 2020 Feb 27;13(1):112. PubMed PMID: 32103773. PMCID: PMC7045487. Epub 2020/02/28. eng.	• Different aim
**90.**	Stang A. Critical evaluation of the Newcastle-Ottawa scale for the assessment of the quality of nonrandomized studies in meta-analyses. Eur J Epidemiol. 2010;25(9):603–5.	• Different aim
**91.**	Stojković B, Igić M, Jevtović Stoimenov T, Tričković Janjić O, Ignjatović A, Kostić M, Petrović M, Stojanović S. Can Salivary Biomarkers Be Used as Predictors of Dental Caries in Young Adolescents? Med Sci Monit. 2020 Jun 10;26:e923471. doi: 10.12659/MSM.923471. PMID: 32518218; PMCID: PMC7301671.	• Different aim
**92.**	Stojković B, Igić M, Jevtović Stoimenov T, Tričković Janjić O, Ignjatović A, Kostić M. Can salivary biomarkers be used as predictors of dental caries in young adolescents? *Med Sci Monitor*. 2020;26:e92347. https://doi.org/10.12659/MSM.923471	• Different aim
**93.**	Subhadharsini S. Serum zinc level in dental caries patients. International Journal of Pharmaceutical Sciences Review and Research. 2015;35(1):19-20.	• Different aim
**94.**	Syed M, Sachdev V, Chopra R. Intercomparison of salivary nitric oxide as a biomarker of dental caries risk between caries-active and caries-free children. Eur Arch Paediatr Dent. 2016 Aug;17(4):239-43. doi: 10.1007/s40368-016-0234-z. Epub 2016 Jun 29. PMID: 27357363.	• Different aim
**95.**	Tao R, Jurevic RJ, Coulton KK, Tsutsui MT, Rob- erts MC, Kimball JR, et al. Salivary antimicro- bial peptide expression and dental caries ex- perience in children. Antimicrob Agents Che- mother. 2005;49(9):3883–8.	• Study on the evaluation of deciduous teeth
**96.**	Tenuta LM, Lima JE, Cardoso CL, Tabchoury CP, Cury JA. Effect of plaque accumulation and salivary factors on enamel demineralization and plaque composition in situ. Pesqui Odontol Bras. 2003 Oct-Dec;17(4):326-31. PubMed PMID: 15107914. Epub 2004/04/27. eng.	• Different aim
**97.**	Thaweboon B, Thaweboon S, Sopavanit C, Kasetsuwan R. A modified dip-slide test for microbiological risk in caries assessment. Southeast Asian J Trop Med Public Health. 2006 Mar;37(2):400-4. PubMed PMID: 17125006. Epub 2006/11/28. eng.	• Different aim<br>• Study on the evaluation of deciduous teeth
**98.**	Thaweboon S, Thaweboon B, Nakornchai S, Jitmaitree S. Salivary secretory IgA, pH, flow rates, mutans streptococci and Candida in children with rampant caries. Southeast Asian J Trop Med Public Health. 2008 Sep;39(5):893-9. PubMed PMID: 19058586. Epub 2008/12/09. eng.	• Study on the evaluation of deciduous teeth
**99.**	Thaweboon S, Thaweboon B, Soo-Ampon S, Soo-Ampon M. Salivary mutans streptococci and lactobacilli after self arresting caries	• Different aim

	treatment. Southeast Asian J Trop Med Public Health. 2005 May;36(3):765-8. PubMed PMID: 16124453. Epub 2005/08/30. eng.	
**100.**	Tikhonova S, Booij L, D'Souza V, Crosara KTB, Siqueira WL, Emami E. Investigating the association between stress, saliva and dental caries: a scoping review. BMC Oral Health. 2018 Mar 13;18(1):41. PubMed PMID: 29534715. PMCID: PMC5851323. Epub 2018/03/15. eng.	• Review
**101.**	Tong H, Gao X, Dong X. Streptococcus oligofermentans sp. nov., a novel oral isolate from caries-free humans. Int J Syst Evol Microbiol. 2003 Jul;53(Pt 4):1101-4. PubMed PMID: 12892133. Epub 2003/08/02. eng.	• Different aim
**102.**	Toro E, Nascimento MM, Suarez-Perez E, Burne RA, Elias-Boneta A, Morou-Bermudez E. The effect of sucrose on plaque and saliva urease levels in vivo. Arch Oral Biol. 2010 Mar;55(3):249-54. PubMed PMID: 20096398. PMCID: PMC2853032. Epub 2010/01/26. eng.	• Different aim:<br>• Study on the evaluation of deciduous teeth
**103.**	van Gelder S, Rohrig N, Schattenberg F, Cichocki N, Schumann J, Schmalz G, et al. A cytometric approach to follow variation and dynamics of the salivary microbiota. Methods. 2018 Feb;134:67-79. PubMed PMID: WOS:000427807500009.	• Different aim
**104.**	Van Nieuw Amerongen A, Bolscher JGM, Veerman ECI. Salivary proteins: Protective and diagnostic value in cariology? Caries Research. 2004;38(3):247-53.	• Conference paper
**105.**	Van Palenstein Helderman WH, Mikx FHM, Van't Hof MA, Truin GJ, Kalsbeek H. The value of salivary bacterial counts as a supplement to past caries experience as caries predictor in children. European Journal of Oral Sciences. 2001;109(5):312-5.	• Different aim<br>• Study on the evaluation of deciduous teeth
**106.**	Van Ruyven FOJ, Lingstrom P, Van Houfe J, Kent R. Relationship among mutons streptococci, low-ph bacteria, and lodophilic polysaccharide-producing bacteria in dental plaque and early enamel caries in humans. Journal of Dental Research. 2000;79(2):778-84.	• Different aim
**107.**	Ventura TMD, Cassiano LDS, Silva C, Taira EA, Leite AD, Rios D, et al. The proteomic profile of the acquired enamel pellicle according to its location in the dental arches. Archives of Oral Biology. 2017 Jul;79:20-9. PubMed PMID: WOS:000401678700004.	• Different aim
**108.**	Vieira AR, Hiller NL, Powell E, Kim LHJ, Spirk T, Modesto A, et al. Profiling microorganisms in whole saliva of children with and without dental caries. Clinical and Experimental Dental Research. 2019;5(4):438-46.	• Different aim<br>• Study on the evaluation of deciduous teeth
**109.**	Vitorino R, Calheiros-Lobo MJ, Duarte JA, Domingues PM, Amado FML. Peptide profile of human acquired enamel pellicle using MALDI tandem MS. Journal of Separation Science. 2008 Feb;31(3):523-37. PubMed PMID: WOS:000253689700011.	• Different aim
**110.**	Vitorino R, Calheiros-Lobo MJ, Williams J, Fer- rer-Correia AJ, Tomer KB, Duarte JA, et al. Peptidomic analysis of human acquired enamel pellicle. Biomed Chromatogr. 2007; 21(11):1107–17.	• Different aim
**111.**	Vitorino R, de Morais Guedes S, Ferreira R, Lobo MJC, Duarte J, Ferrer Correia AJ, et al. Two dimensional electrophoresis study of in vitro pellicle formation and dental caries suscepti- bility. Eur J Oral Sci. 2006;114(2):147–53.	• Different aim
**112.**	Vlková B, Celec P. Does Enterococcus faecalis contribute to salivary thiobarbituric acid-reacting substances? In Vivo. 2009;23(2):343-6.	• Different aim

**113.**	Voelker MA, Simmer-Beck M, Cole M, Keeven E, Tira D. Preliminary findings on the correlation of saliva pH, buffering capacity, flow, Consistency and Streptococcus mutans in relation to cigarette smoking. J Dent Hyg. 2013 Feb;87(1):30-7. PubMed PMID: 23433696. Epub 2013/02/26. eng.	• Different aim
**114.**	Wang K, Wang X, Zheng S, Niu Y, Zheng W, Qin X, Li Z, Luo J, Jiang W, Zhou X, Li W, Zhang L. iTRAQ-based quantitative analysis of age-specific variations in salivary proteome of caries-susceptible individuals. J Transl Med. 2018 Oct 25;16(1):293. doi: 10.1186/s12967-018-1669-2. PMID: 30359274; PMCID: PMC6202833.	• Different aim
**115.**	Wang K, Wang Y, Wang X, Ren Q, Han S, Ding L, et al. Comparative salivary proteomics analysis of children with and without dental caries using the iTRAQ/MRM approach. J Transl Med. 2018;16(1):11–3.	• Study on the evaluation of deciduous teeth
**116.**	Wang K, Zhou X, Li W, Zhang L. Human salivary proteins and their peptidomimetics: Values of function, early diagnosis, and therapeutic po- tential in combating dental caries. Arch Oral Biol. 2019;99:31–42.	• Review
**117.**	Wang ZY, Wang JQ, Zhou Y, Zhao D, Xiao B. Quantitative detection of Streptococcus mutans and bacteria of dental caries and no caries groups in permanent teeth from a north China population. Chin Med J (Engl). 2012 Nov;125(21):3880-4. PubMed PMID: 23106892. Epub 2012/10/31. eng.	• Different aim
**118.**	Watanabe Y, Mizoguchi H, Masamura K, Nagaya T. No relationship of salivary flow rate or se-cretory immunoglobulin A to dental caries in children. Environ Health Prev Med. 1997; 2(3):122–5.	• Study on the evaluation of deciduous teeth
**119.**	Whitford GM, Wasdin JL, Schafer TE, Adair SM. Plaque fluoride concentrations are dependent on plaque calcium concentrations. Caries Res. 2002 Jul-Aug;36(4):256-65. PubMed PMID: 12218274. Epub 2002/09/10. eng.	• Different aim
**120.**	Wolff MS, Larson C. The cariogenic dental biofilm: good, bad or just something to control? Braz Oral Res. 2009;23 Suppl 1:31-8. PubMed PMID: 19838556. Epub 2009/10/27. eng.	• Different aim
**121.**	Xingqun C, Xuedong Z, Xin X. [Application of saliva in disease diagnosis]. Hua Xi Kou Qiang Yi Xue Za Zhi. 2016 Dec 1;34(6):647-653. Chinese. doi: 10.7518/hxkq.2016.06.019. PMID: 28318170; PMCID: PMC7030871.	• Review
**122.**	Xu L, Chen X, Wang Y, Jiang W, Wang S, Ling ZX, et al. Dynamic Alterations in Salivary Microbiota Related to Dental Caries and Age in Preschool Children With Deciduous Dentition: A 2-Year Follow-Up Study. Frontiers in Physiology. 2018 Apr;9. PubMed PMID: WOS:000429193500001.	• Different aim:<br>• Study on the evaluation of deciduous teeth
**123.**	Xu Y, Jia YH, Chen L, Huang WM, Yang DQ. Metagenomic analysis of oral microbiome in young children aged 6–8 years living in a rural isolated Chinese province. Oral Diseases. 2018;24(6):1115-25.	• Different aim<br>• Study on the evaluation of deciduous teeth
**124.**	Yamamoto Y, Washio J, Shimizu K, Igarashi K, Takahashi N. Inhibitory Effects of Nitrite on Acid Production in Dental Plaque in Children. Oral Health & Preventive Dentistry. 2017 Apr;15(2):153-6. PubMed PMID: WOS:000400667100008.	• Different aim<br>• Study on the evaluation of deciduous teeth
**125.**	Yang Y, Li YH, Lin YH, Du MQ, Zhang P, Fan MW. Comparison of immunological and microbiological characteristics in children and the elderly with or without dental caries. European Journal of Oral	• Study on the evaluation of deciduous teeth

	Sciences. 2015 Apr;123(2):80-7. PubMed PMID: WOS:000351166700004.	
**126.**	Zabokova Bilbilova E, Sotirovska Ivkovska A, Georgiev Z, Stefanovska E. Evaluation of buffer capacity of saliva in caries-free and caries-active children. Pril (Makedon Akad Nauk Umet Odd Med Nauki). 2013;34(2):151-7. PubMed PMID: 24280889. Epub 2013/11/28. eng.	• Different aim<br>• Study on the evaluation of deciduous teeth
**127.**	Zarco MF, Vess TJ, Ginsburg GS. The oral microbiome in health and disease and the potential impact on personalized dental medicine. Oral Dis. 2012 Mar;18(2):109-20. PubMed PMID: 21902769. Epub 2011/09/10. eng.	• Different aim
**128.**	Zhang CZ, Cheng XQ, Li JY, Zhang P, Yi P, Xu X, et al. Saliva in the diagnosis of diseases. Int J Oral Sci. 2016 Sep 29;8(3):133-7. PubMed PMID: 27585820. PMCID: PMC5113094. Epub 2016/09/03. eng.	• Review
**129.**	Zhang X, Kulasinghe A, Karim RS, Punyadeera C. Saliva diagnostics for oral diseases. Advances in Salivary Diagnostics2015. p. 131-56.	• Review
**130.**	Zhang YF, Li DY, Yu JX, He HT. On the thickness and nanomechanical properties of salivary pellicle formed on tooth enamel. J Dent. 2016 Dec;55:99-104. PubMed PMID: 27771252. Epub 2016/10/26. eng.	• Different aim
**131.**	Zhao A, Blackburn C, Chin J, Srinivasan M. Soluble toll like receptor 2 (TLR-2) is increased in saliva of children with dental caries. BMC Oral Health. 2014 Aug 31;14:108. PubMed PMID: 25174416. PMCID: PMC4236648. Epub 2014/09/02. eng.	• Study on the evaluation of deciduous teeth
**132.**	Zhou J, Jiang N, Wang S, Hu X, Jiao K, He X, et al. Exploration of human salivary microbiomesâ€"insights into the novel characteristics of microbial community structure in caries and caries-free subjects. PLoS ONE. 2016;11(1).	• Different aim
**133.**	Zhou JY, Zhu YJ, Yu ZH, Jiao KL, Wu F, Li ZQ. Microbial community structure in saliva from children of the Baoan nationality with or without dental caries in gansu, China. Biomedical Research-India. 2017;28(1):211-6. PubMed PMID: WOS:000393483400036.	• Study on the evaluation of deciduous teeth
**134.**	Żyła T, Kawala B, Antoszewska-Smith J, Kawala M. Black stain and dental caries: a review of the literature. Biomed Res Int. 2015;2015:469392. PubMed PMID: 25802850. PMCID: PMC4354720. Epub 2015/03/25. eng.	• Review of literature
